# Prospect of research hotspots in prevention and treatment of diseases based on intestinal microbiome

**DOI:** 10.3389/fimmu.2022.971457

**Published:** 2022-09-14

**Authors:** Yue Cai, Yuting Zhang, Wenxue Wang, Jiawei Geng

**Affiliations:** ^1^ The Affiliated Hospital of Kunming University of Science and Technology. Department of Infectious Disease and Hepatic Disease, The First People's Hospital of Yunnan Province, Kunming, Yunnan, China; ^2^ Department of Infectious Disease and Hepatic Diseases, The First People’s Hospital of Yunnan Province, The Affiliated Hospital of Kunming University of Science and Technology, Kunming, China

**Keywords:** gut microbiota, metabolite, fecal microbiota transplantation, gene editing, precise therapy

## Abstract

With the in-depth study of gut microbiota, the methods of preventing and treating diseases have gradually diversified. But there is still lack of precise therapies methods to better treat the diseases. Therefore, researcher must focus on how to accurately regulate gut microbiota to achieve it. In order to promote the rapid development of this field, we provide several insights in gut microbiome-based precision therapies while prospecting the future directions.

## Introduction

With the deepening of intestinal microbiome research, the methods of intervention and regulation of intestinal microbiome have diversified. From traditional fecal microbiota transplantation (FMT) to probiotics, prebiotics, synbiotics and postbiotics, to gene editing therapies targeting intestinal microbiome, the intestinal microbiome field is booming.

Because intestinal symbiotic bacteria play an important role in health and disease, research based on the intestinal microbiome has become one of the most active frontier fields in current biomedicine. However, when it comes to turning “in-body gut bacteria” into drugs, we have long been limited to fecal microbiota transplantation (FMT). FMT aims to address the problem of dysbiosis by transferring fecal microbiota from healthy donors into patients.

Although FMT is known to have had considerable success in treating Clostridium difficile infection (CDI) and has shown some efficacy in treating some other diseases (irritable bowel syndrome, inflammatory bowel disease, cardiovascular disease, metabolic diseases and so on) as well, the exact mechanisms of its efficacy are poorly understood. Furthermore, since FMT is a “bacterial hodgepodge” therapy, it is not repeatable and has safety risks. In particular, FMT is very easy to cause the transmission of antibiotic-resistant bacteria or antibiotic-resistant genes. Thus, therapies based on gut microbiota need to be more targeted.

In order to find gut microbiome-based precision therapies to prevent and treat diseases, the following aspects will be the future research directions: Firstly, we need to first identify which types of gut bacteria are missing or overgrowing to cause a disease, so as to purposefully supplement or reduce this type of gut bacteria. In a study on colitis, inflammation is usually associated with microbiota dysregulation, mainly manifested by changes in gut microbiota, the most important of which is the increase of facultative anaerobe Enterobacteriaceae. In mouse models of colitis, tungstate selectively prevented Enterobacteriaceae from proliferating in the intestine, but nearby beneficial bacteria were unaffected (1). With further research they found that tungstate can inhibits microbial respiratory pathways that function only during episodes of inflammation, thereby preventing or treating adverse intestinal effects caused by microbiota dysbiosis through precise editing of microbiota composition (1). Thus, This suggests that it is possible to achieve positive therapeutic outcomes for the host by precisely manipulating a particular type of intestinal flora and thereby altering the function and composition of the intestinal microbiota.

Secondly, when we treat a disease with a drug, we find that some patients are responders and some patients are non-responders. This requires determining which types of bacteria are responsible for the drug’s therapeutic effect. A first supplement of this type of gut bacteria, followed by drug treatment, would be more effective in those who do not respond. In some cancer treatments, inosine modulates anti-tumor immunity checkpoint blockade immunotherapy, which can harness the immune system to kill cancer cells, but is only effective in certain patients who have unique beneficial bacteria in their gut. This is because the unique bacteria can produce a metabolite, that is inosine, which greatly enhances the effect of this treatment. According to a study, in four mouse models of cancer, the researchers isolated three bacteria species that produce inosine– *Bifidobacterium pseudolongum*, *Lactobacillus Johnsonii* and *Olsenella*, which noteworthy increased the efficacy of checkpoint inhibitor immunotherapy (2). They also found that in the presence of proinflammatory stimuli and immunotherapy, inosine strongly enhances the anticancer ability of T cells to attack tumors in a variety of tumor types, including colorectal, bladder, and melanoma. And even shrinking the tumor and eliminating colorectal cancer cells in some cases eventually (2). Thus, this suggests that certain bacteria in the gut may increase the efficacy of immunotherapy, making it possible for microbial therapy to be incorporated into immunotherapy to better treat cancer.

Thirdly, if gene expression in certain gut bacteria causes disease, we can use gene editing techniques to target and silence the expression of these genes. But the precise addition or removal of microbial strains or genes from complex communities has not been solved, limiting the full role of the gut microbiome in disease susceptibility and treatment. Recently, Peter Turnbaugh’s team has achieved CRISPR-Cas9-based technology to regulate the microbiome by targeting one bacterial strain or gene at a time (3). This is the first stable gene editing in the mammals that altered the genomes of gut bacteria. The researchers used a engineered bacteriophage called M13 to deliver DNA and to inject the CRISPR-Cas9 system into the cells of a specific *Escherichia coli* strain and then started cutting DNA fragments and removed large chunks of genes. It also changes the overall composition of the bacterial community in the digestive system (3). This means that stable gene editing has been achieved in the mammalian gut microbiome, which may be the starting point for attempts to engineer gut bacteria. Gene editing technology allows us to precisely target harmful strains of bacteria in the gut microbiome while keeping beneficial strains undisturbed. It can allow us to study the microbiome in a more controlled way than before, and thus go on to treat diseases more accurately and effectively.

Last but not least, we also need to identify metabolites and components of intestinal bacteria that are implicated in disease. That is, to promote the study of postbiotics. As signal molecules and substrates of host metabolic reactions, gut microbiota metabolites affect host physiology, pathology and other processes. Among them, short-chain fatty acids (SCFAs) are the most commonly studied class of small-molecule metabolites, which are produced by the fermentation of dietary fiber by gut microbes. Recent studies have shown that SCFAs can modulate host physiological and biochemical functions, including maintenance of native gut barrier function, gut motility, gut hormone secretion, chromatin regulation, gut-brain axis, immune function, etc. (4).

Collectively, understanding these microbial signatures and the mechanisms behind them could eventually lead to new therapies. One day, it is hoped that by selecting specific strains of bacteria in the gut, or even just individual genes that you want to promote or eliminate, could be used precisely to facilitate the growth of beneficial gut bacteria in humans. The regulation and intervention of gut microbes are of great significance in the treatment of various diseases. Although the current medical application of microbiota-based therapy is in infancy, its potential to transform medical practice is considerable, including the potential to control antibiotic use, and also to augment or replace the existing therapies in the areas of cardiovascular medicine, neurology and psychiatry. In the future, therapeutic intervention for a specific type of microbes will be one of the hot areas for precise and personalized treatment of diseases.

## Data availability statement

The original contributions presented in the study are included in the article/supplementary material, further inquiries can be directed to the corresponding author/s.

## Author Contributions

YC collected the data and wrote most of the manuscript with help from YZ, WW, JG. All authors contributed to the article and approved the submitted version.

## Funding

This work was supported by the National Natural Science Foundation of China (Grant No. 81860437) and the Yunnan Province Innovation Team of Intestinal Microecology-Related Disease Research and Technological Transformation (Grant No. 202005AE160010).

## Conflict of interest

The authors declare that the research was conducted in the absence of any commercial or financial relationships that could be construed as a potential conflict of interest.

## Publisher’s note

All claims expressed in this article are solely those of the authors and do not necessarily represent those of their affiliated organizations, or those of the publisher, the editors and the reviewers. Any product that may be evaluated in this article, or claim that may be made by its manufacturer, is not guaranteed or endorsed by the publisher.
